# Tracing the colonization history of the Indian Ocean scops-owls (Strigiformes: *Otus*) with further insight into the spatio-temporal origin of the Malagasy avifauna

**DOI:** 10.1186/1471-2148-8-197

**Published:** 2008-07-09

**Authors:** Jérôme Fuchs, Jean-Marc Pons, Steven M Goodman, Vincent Bretagnolle, Martim Melo, Rauri CK Bowie, David Currie, Roger Safford, Munir Z Virani, Simon Thomsett, Alawi Hija, Corinne Cruaud, Eric Pasquet

**Affiliations:** 1 UMR5202 «Origine, Structure et Evolution de la Biodiversité», Département Systématique et Evolution, Muséum National d'Histoire Naturelle, 55 Rue Buffon, 75005 Paris, France; 2 Service Commun de Systématique Moléculaire, IFR CNRS 101, Muséum National d'Histoire Naturelle, 43, rue Cuvier, 75005 Paris, France; 3 DST-NRF Centre of Excellence at the Percy FitzPatrick Institute, University of Cape Town, Rondebosch 7701, Cape Town, Republic of South Africa; 4 Museum of Vertebrate Zoology and Department of Integrative Biology, 3101 Valley Life Science Building, University of California, Berkeley, CA 94720-3160, USA; 5 Field Museum of Natural History, 1400 South Lake Shore Drive, Chicago, IL 60605, USA; 6 Vahatra, BP3972, Antananarivo (101), Madagascar; 7 CEBC-CNRS, Beauvoir sur Niort, 79360, France; 8 UMR5175 Centre d'Ecologie Fonctionnelle et Evolutive, 1919 Route de Mende, F-34293 Montpellier Cedex 5, France; 9 Nature Seychelles, PO Box 1310, Victoria, Mahé, Republic of Seychelles; 10 BirdLife International, Wellbrook Court, Girton Road, Cambridge CB3 0NA, UK; 11 The Peregrine Fund, 5668 West Flying Hawk Lane, Boise Idaho 83709, USA; 12 Department of Biology, Leicester University, LE1 7RH, UK; 13 Department of Ornithology, National Museums of Kenya, P.O. Box 40658, Nairobi, Republic of Kenya; 14 Department of Environment, Ministry of Agriculture, Natural Resources, Environment and Cooperatives, Zanzibar Revolutionary Government, P.O. Box 811, Zanzibar, United Republic of Tanzania; 15 Genoscope, Centre National de Séquençage, 2 rue Gaston Crémieux, CP5706, 91057 Evry Cedex, France

## Abstract

**Background:**

The island of Madagascar and surrounding volcanic and coralline islands are considered to form a biodiversity hotspot with large numbers of unique taxa. The origin of this endemic fauna can be explained by two different factors: vicariance or over-water-dispersal. Deciphering which factor explains the current distributional pattern of a given taxonomic group requires robust phylogenies as well as estimates of divergence times. The lineage of Indian Ocean scops-owls (*Otus*: Strigidae) includes six or seven species that are endemic to Madagascar and portions of the Comoros and Seychelles archipelagos; little is known about the species limits, biogeographic affinities and relationships to each other. In the present study, using DNA sequence data gathered from six loci, we examine the biogeographic history of the Indian Ocean scops-owls. We also compare the pattern and timing of colonization of the Indian Ocean islands by scops-owls with divergence times already proposed for other bird taxa.

**Results:**

Our analyses revealed that Indian Ocean islands scops-owls do not form a monophyletic assemblage: the Seychelles *Otus insularis *is genetically closer to the South-East Asian endemic *O. sunia *than to species from the Comoros and Madagascar. The Pemba Scops-owls *O. pembaensis*, often considered closely related to, if not conspecific with *O. rutilus *of Madagascar, is instead closely related to the African mainland *O. senegalensis*. Relationships among the Indian Ocean taxa from the Comoros and Madagascar are unresolved, despite the analysis of over 4000 bp, suggesting a diversification burst after the initial colonization event. We also highlight one case of putative back-colonization to the Asian mainland from an island ancestor (*O. sunia*). Our divergence date estimates, using a Bayesian relaxed clock method, suggest that all these events occurred during the last 3.6 myr; albeit colonization of the Indian Ocean islands were not synchronous, *O. pembaensis *diverged from *O. senegalensis *about 1.7 mya while species from Madagascar and the Comoro diverged from their continental sister-group about 3.6 mya. We highlight that our estimates coincide with estimates of diversification from other bird lineages.

**Conclusion:**

Our analyses revealed the occurrence of multiple synchronous colonization events of the Indian Ocean islands by scops-owls, at a time when faunistic exchanges involving Madagascar was common as a result of lowered sea-level that would have allowed the formation of stepping-stone islands. Patterns of diversification that emerged from the scops-owls data are: 1) a star-like pattern concerning the order of colonization of the Indian Ocean islands and 2) the high genetic distinctiveness among all Indian Ocean taxa, reinforcing their recognition as distinct species.

## Background

The island of Madagascar is considered a biodiversity hotspot with an intriguing endemic fauna [1]. The origin of the island's peculiar and highly unique fauna can be explained by two contrasting processes: vicariance when Madagascar became separated from the African landmass approximately 165 mya and from India approximately 88 mya [2,3], or over-water dispersal from the African, Australian and Eurasian landmasses. In near proximity to Madagascar are several archipelagos, which have different geological histories. The eastern islands in the Seychelles archipelago are of granitic origin and likely broke off from India and reached their current position some 55–75 mya when India drifted northwards [4], while the western portion of the Seychelles is comprised of recent atolls. In contrast, the volcanic Comoros archipelago is of relatively recent age (0–11 mya), hence the only plausible explanation for the colonization of its biota is by over-ocean dispersal from Africa, Australia, Madagascar or Eurasia.

The origin, timing and modes (vicariance versus over-water dispersal) of colonization by animals of Madagascar and in some cases the surrounding Comoro islands have been the focus of several research programs [5-10]. Given the antiquity of the break-up of Indo-Madagascar from Africa, which considerably pre-dates the first known fossil records of most modern families or genera of animals, in particular vertebrates, it is now assumed that the majority of extant groups arrived on Madagascar via over-water dispersal [11]. From recent phylogenetic studies, as well as traditional taxonomy, it is inferred that most of the Malagasy avifauna originated from African ancestors, although colonization events from Eurasia and Australia have also been documented for flying organisms such as bats and birds [12-16].

Interpreting the origin of certain components of the Malagasy avifauna is in many cases difficult when based on current taxonomical classifications alone as several genera of birds are shared between Madagascar, Africa and Eurasia. Among these are scops-owls of the genus *Otus*. As currently defined, *Otus *is present in five biogeographic areas (Indo-Malaya, Afrotropics, Nearctic, Neotropics, Palearctic) [17]. However, monophyly of *Otus *(sensu [17]) is uncertain; some molecular studies suggested that the New World *Otus *(*Megascops*-including *Otus flammeolus*, see [18]), which differ from Old World *Otus *by song type, are genetically more closely related to the widespread owl genera *Strix *and *Bubo *[19,20] whereas the African White-faced Owl (*O. leucotis*) has closer affinities with the genus *Asio *[20]. The above-mentioned taxa excluded, *Otus *species have their center of diversity in Eurasia (26 species); secondary radiations occur in the Indian Ocean (six or seven species) and Africa (four species). To our knowledge, the earliest known *Otus *fossil, a distal end of the right humerus, is from western Kenya and dates from the Miocene (16.5–18.5 mya; [21]). This partial fossil is morphologically close to *O. senegalensis*, but its relationship among members of the genus is not clear. The western Indian Ocean taxa are often thought to constitute a superspecies (*rutilus *group) of five or six species [22]: *O. capnodes *from Anjouan, *O. mayottensis *from Mayotte, *O. moheliensis *from Mohéli, *O. pauliani *from Grande Comore, and *O. rutilus*/*O. madagascariensis *from Madagascar (see [23] for a discussion about phylogeography and taxonomic status of the two Malagasy forms), and *O. insularis *from the granitic Seychelles. The latter species shows apparent affinities, based on vocalization data, to the Indonesian *O. magicus *[24]. Most of the western Indian Ocean taxa are poorly known: specific status has been proposed only within the last twenty years for *Otus pauliani*, *O. capnodes*, *O. madagascariensis *and *O. mayottensis *using both biometric and vocalization data [25-28], while *O. moheliensis *was first described in 1998 [29].

The evolutionary and biogeographic history of the Indian Ocean *Otus *taxa has not been the focus of a phylogenetic study. In this paper, we use two nuclear introns (myoglobin intron-2 and TGFB2 intron-5) and four mitochondrial protein coding-genes (ND2, ND3, ATP6, cytochrome-*b*) in order to propose a first multi-locus phylogeny of scops-owls and to track their colonization history of the western Indian Ocean islands in space and time. We additionally compare the biogeographic affinities of Malagasy scops-owl species to those of other Malagasy avian lineages which have been the focus of recent genetic studies [12,13,16].

## Results

### Sequence properties

We obtained between 680 and 726 bp (*Otus lettia ussuriensis *and *Aegolius acadicus*, respectively) for the myoglobin intron-2 resulting in a final alignment of 749 bp. Among the 749 bp, 151 were variable (20%) and 56 were parsimony informative (7.5%). Maximum Likelihood (ML) analyses yielded one tree (-ln = 2198.81) that slightly differs topologically from the 50% majority consensus rule tree obtained from the Bayesian analyses (-ln = 2442.98). We obtained between 561 and 593 bp (*O. leucotis *and *O. pembanesis*/*O. senegalensis *Allele 1, respectively) for the TGFB2 intron-5 resulting in a final alignment of 605 bp. Three individuals were found to be length-variant heterozygotes: *O. leucotis *possesses a CCT duplication in a region with a CCT pattern in all other species, *O. rutilus *(FMNH 431150) possesses a one base pair deletion (G) in position 171 of the alignment, and *O. senegalensis *possesses a one base pair insertion (A in position 559 of our alignment; this insertion was also found in the two *O. pembaensis *individuals sequenced). The two *O. senegalensis *alleles also differ by two further mutations; these two alleles clustered together as the sister-group to *O. pembaensis *in a ML analysis (tree not shown). Therefore, we use the consensus sequence (the two single nucleotide polymorphisms were coded using the appropriate IUPAC code) from the two *O. senegalensis *alleles for further phylogenetic analyses. Only the alleles without the insertion/deletion were included in the phylogenetic analyses for *O. leucotis *and *O. rutilus*, as the insertion/deletion events were autapomorphic in both cases. Among the 601 base pairs retained for the analyses, 163 were variable (27%) and 71 were parsimony informative (11.8%). ML analyses yielded one tree (-ln = 2131.71) that slightly differs from the 50% majority consensus rule tree obtained from the Bayesian analyses (-ln = 2179.36).

**Figure 1:**
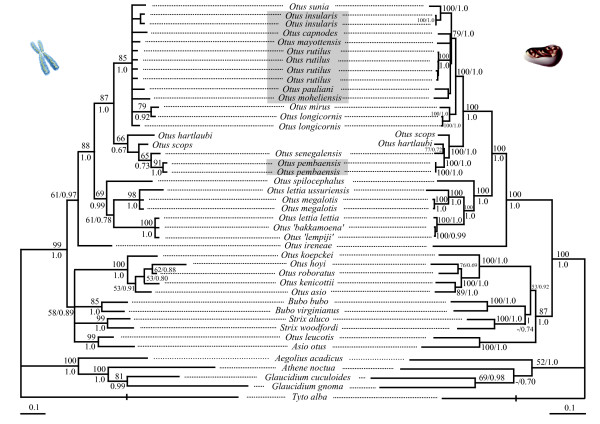
Fifty percent majority-rule consensus tree resulting from the Bayesian mixed-model analyses of the nuclear (left, arithmetic mean, -ln = 4638.29) and mitochondrial (right, arithmetic mean, -ln = 27725.63) data sets. Values close to nodes represent MP bootstrap percentages and BI posterior probabilities. Grey blocks highlight Indian Ocean taxa. Species between quotes indicate samples for which geographic origin is unknown (captive individuals). Branch lengths of the outgroup (*Tyto alba*) were reduced by a scale of two for graphical purpose.

The topologies obtained from the nuclear loci were very similar to each other, delineating the primary clades without achieving resolution at the tips (see Additional Files 1 and 2). The 50% majority consensus rule tree obtained from the Bayesian analyses (Figure 1, -ln = 4638.29) and Maximum Parsimony strict consensus tree (105675 equally parsimonious trees of 412 steps, CI = 0.82, RI = 0.86) of the two concatenated nuclear loci provided a well-resolved topology for inter-generic relationships as well as some resolution of relationships among the primary *Otus *lineages but failed to provide resolution among members of the Indian Ocean radiation.

The concatenated mitochondrial sequences retained for analyses were 2983 bp long (1047 bp for ND2, 684 bp for ATP6, 351 bp for ND3 and 901 bp for cytochrome-*b*) and correspond to the positions 5246 to 6281 (ND2), 9240 to 9923 (ATP6), 10776 to 11120 (ND3), and 15011 to 15911 (cytochrome-*b*) of the *Gallus gallus *mitochondrial genome sequence [30]; GenBank accession number X52392). The ATP6 and cytochrome-*b *sequences contained no insertions, deletions and stop-codons in the reading frame. The ND2 sequence of *Aegolius acadicus *exhibits a two-codon insertion (CAA ACC) just before the stop codon. All the ND3 sequences exhibited the pyrimidine insertion (T for *O. capnodes*, C for all other species analyzed) previously reported for several clades of birds [31]; this extra-nucleotide was removed before phylogenetic analyses. Partitioning the gene by codon positions significantly improved the fit of models to the data for all four mitochondrial loci, as inferred from the Bayes Factor (B_F_) values (BF_ND2 _= 826.3, BF_ATP6 _= 681.7, BF_ND3 _= 319.1, BF_cytb _= 1048.8). Mitochondrial gene trees were very similar to each other (ND2: ML -ln = 10556.45, BI partitioned by codon position = 10161.23 – Additional File 3; ATP6: ML -ln = 6550.30, BI partitioned by codon position = 6232.87 – Additional File 4; ND3: ML -ln = 3266.29, BI partitioned by codon position -ln = 3146.47 – Additional File 5; cytochrome-*b*, ML -ln = 8623.79, BI partitioned by codon position -ln = 8128.06 – Additional File 6), albeit levels or resolution varied among genes. As expected, no conflict was detected between the individual evolutionary histories of the mitochondrial gene trees (as inferred from posterior probabilities). The 50% majority-rule tree obtained from the concatenated analyses of the mitochondrial genes (partitioned by gene and codon position: -ln = 27725.63, Figure 1) was very similar in terms of topology and number of supported nodes to the MP strict consensus tree (two equally most parsimonious trees of 5922 steps, CI = 0.42, RI = 0.60).

### Phylogenetic results

**Figure 2:**
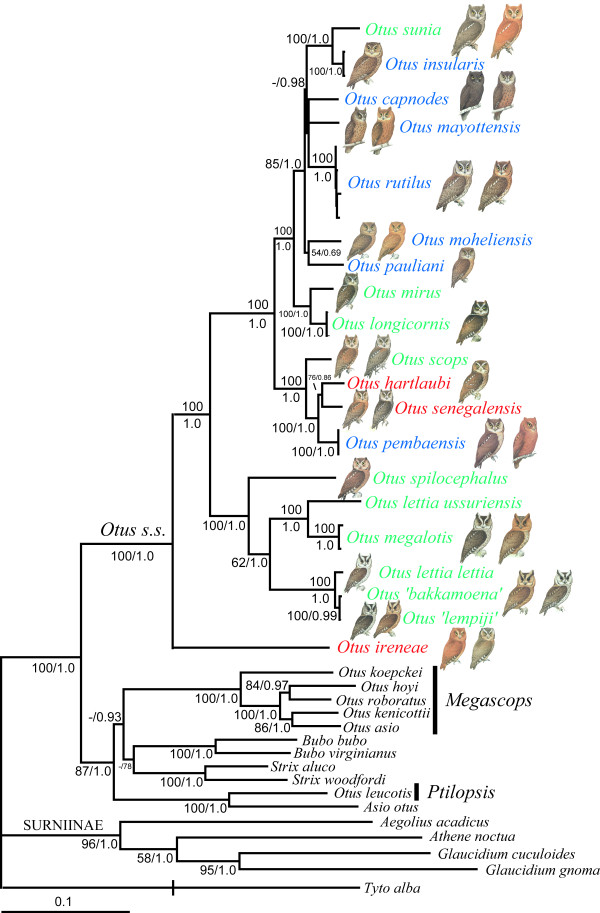
Fifty percent majority-rule consensus tree resulting from the mixed-model analyses of the concatenated data set (14 partitions, arithmetic mean, -ln = 32195.91). Values close to nodes represent MP bootstrap percentages and BI posterior probabilities. Note the occurrence of color morphs in most of the *Otus sensu stricto *species. Colors for *Otus *taxa names refer to geographic distribution (green: South-East Asia, red: Africa and Blue: Indian Ocean Islands). The genera *Ptilopsis *(African White-faced Owl) and *Megascops *(New World Screech Owls) refer to taxa that were previously included in *Otus*. Species between quotes indicate samples for which geographic origin is unknown (captive individuals). The branch length of the outgroup (*Tyto alba*) was reduced by a scale of two for graphical purpose. Pictures were modified from [32].

The individual trees obtained from the two nuclear introns and mitochondrial data sets were very similar to each other and no incongruence was detected between the nuclear and mitochondrial topologies (Figure 1), according to the criteria defined in the Material and Methods section. Further, there was usually strong congruence for nodal support among the different methods. Most of the nodes present in the 50% majority-rule consensus tree resulting from the Bayesian analyses performed on the concatenated data set (partitioned by gene and codon position- 14 partitions-, -ln = 32195.91, Figure 2) were very well supported in the parsimony analyses too (two equally most parsimonious trees, 6340 steps, CI = 0.44, RI = 0.62).

The African species *O. ireneae *was recovered as the sister-taxon of all remaining *Otus sensu stricto *scops-owls. All the western Indian Ocean taxa clustered in a clade that also contains the Eurasian *O. scops*, the São Tomé endemic *O. hartlaubi*, the Pemba Island endemic *O. pembaensis*, the African mainland *O. senegalensis*, the Philippine taxa *O. mirus *and *O. longicornis*, as well as the Indo-Malayan *O. sunia*. The western Indian Ocean taxa were not recovered as a monophyletic lineage since the Seychelles *O. insularis *was more closely related to the Indo-Malayan *O. sunia *than to any other taxon occurring in the western Indian Ocean region. Uncorrected-p mitochondrial distances among members of the Indian Ocean taxa/*O. sunia *clade range between 4.6% (between *O. capnodes *and *O. rutilus*) and 7.0% (*O. sunia *and *O. rutilus*), with a mean of 5.3% (s.d. = 0.7%). Most of the relationships between the Comorian and Malagasy taxa (*capnodes*, *mayottensis*, *moheliensis*, *pauliani*, *rutilus*) did not receive statistical support and short inter-nodes characterized most of the branches among these lineages. We attribute this lack of resolution to rapid speciation events ('hard polytomy') rather than a lack of sufficient character sampling ('soft polytomy') because 1) we sampled several genes with different evolutionary properties resulting in a final alignment of more than 4300 bp and, 2) the nodes above and below the polytomy received bootstrap percentages of 70% or more or posterior probabilities of 0.95 or greater. The remaining *Otus *species clustered in a second large clade. Within this latter clade, *O. lettia ussuriensis *(eastern Russia) did not cluster with another *O. lettia *sample from Laos, but with the Philippine taxon *O. megalotis*, suggesting that further work with more complete geographic sampling is needed to address the evolutionary history of the *O. lettia*/*O. megalotis *species complex.

### Dating analyses

The biogeographic history inferred from the topology of the concatenated analyses is intriguing as several faunistic exchanges involving the Indian Ocean islands and Indo-Malaya region occurred (split *O. mirus-O. longicornis *from the Indian Ocean taxa and split between *O. insularis *and *O. sunia*). These faunistic exchanges imply either multiple colonization events of the western Indian Ocean islands or one re-colonization of the mainland by *O. sunia*, about 0.25–0.30 mya. The main radiation of western Indian Ocean island taxa (2.5 mya, 95% HPD = 1.2–4.0 Table 1) occurred soon after the initial colonization event (3.6 mya, 95% HPD = 1.8–6.0). Our analyses revealed that *O. hartlaubi*, endemic to the African Atlantic island of São Tomé, and *O. pembaensis*, restricted to Pemba Island off the east African coast, have strong affinities with the African mainland species *O. senegalensis*. A very close relationship between *O. senegalensis *and *O. pembaensis *is further supported by the fact that these two taxa share a one nucleotide insertion in the TGFB2 locus. The position of *O. harlaubi *in the mitochondrial and concatenated tree renders this insertion paraphyletic. Yet, it is worth noting that the two alleles of *O. senegalensis *differ in length at this site. Considering that the first colonizers of São Tomé and Pemba were probably in small numbers when compared to the continental and widely distributed *O. senegalensis*, we regard the discrepancy between the mitochondrial and nuclear trees as being due to the different effective population size of the markers (the mitochondrial genome has an effective population size that is one fourth of the nuclear genome) and random processes (coalescence). The basal split within *Otus sensu stricto *occurred about 11.7 mya (95% HPD = 6.0–19.0) and the divergence between the two primary clades about 9.3 mya (95% HPD = 4.7–15.1, Figure 2).

**Table 1:** Posterior distribution of divergence times for some selected nodes.

Calibration point	mya (95% HPD)
*O. sunia*/*O. insularis*	1.1 (0.3–2.0)
*Otus pauliani*/O. moheliensis *	0.7 (0.5–0.9)
*Otus *Indian Ocean radiation	2.5 (1.2–4.0)
*Otus *Indian Ocean colonization	3.6 (1.8–6.0)
Pemba island colonization	1.7 (0.7–3.3)
Clade 1/Clade 2	9.3 (4.7–15.1)
*O. ireneae*/remaining *Otus*	11.7 (6.0–19.0)
Asioninae/sister-group *	17.3 (16.7–19.3)

mya: million years ago. * denote the two calibration points.

## Discussion

### Phylogeny of Otus sensu stricto

Previous studies [18,20] highlighted that several species usually assigned to *Otus (Ptilopsis leucotis, Megascops*) are not directly related to *Otus*. Our analyses, using independent samples and additional genes, confirmed the lack of direct relationships among these three lineages. Given that these results were previously suggested, we do not discuss them further.

Our analyses based on nuclear and mitochondrial sequence data provide the first phylogenetic hypothesis on the diversification of *Otus sensu stricto*. We suggest that *Otus *consists of at least three primary lineages. The first lineage consists of the 'relictual' Sokoke Scops-owl (*O. ireneae*), endemic to the coastal forests of Kenya and Tanzania. This species is sometimes considered to form a superspecies with the patchily distributed Sandy Scops-owl (*O. icterorhynchus*) [22], the only African *Otus *species we were not able to sample.

The second major lineage is restricted to South-East Asia and consists of *O. spilocephalus*, the *O. bakkamoena*-*O. lettia*-*O. lempiji *superspecies, as well as *Otus lettia ussuriensis *and *O. megalotis*. As inferred from our results, the current taxonomy within this group appears to be problematic as *O. lettia lettia *and *O. l. ussuriensis *are not recovered as sister-taxa. *O. l. ussuriensis*, restricted to Sakhalin, Ussuriland and North-East China, is sometimes considered to be related to the Japanese Scops-owl (*O. semitorques*), based on voice, plumage and iris color data [22]. We could not include the latter species in the present work, but highlight here that this relationship needs to be further tested using molecular data.

The third major lineage includes all the remaining *Otus *species we sampled and is divided in two subclades that consist of 1) *O. scops*, *O. senegalensis*, *O. hartlaubi *and *O. pembaensis*, and 2) *O. longicornis*, *O. mirus*, *O. mayottensis*, *O. rutilus*, *O. capnodes*, *O. moheliensis*, *O. pauliani*, *O. insularis *and *O. sunia*. Relationships among these taxa are discussed below.

### Phylogeny and origin of the western Indian Ocean Otus

Data accumulated in recent years on the biology and distribution of the western Indian Ocean scops-owls [26-29] together with our analyses based on DNA sequences of six loci, shed new light on the evolutionary history of these birds. The phylogeny we propose here is in full agreement with recent work based on phenotypic characters suggesting that *O. pauliani*, *O. capnodes*, *O. moheliensis *and *O. mayottensis *represent distinct evolutionary lineages [25,28,29]. In addition we find good support for Asian biogeographic affinities of the western Indian Ocean islands *Otus *spp., and highlight that they may constitute a paraphyletic assemblage.

The Indo-Malayan *O. sunia *formed a well-supported clade with *O. insularis *from the granitic Seychelles, which was very closely related to the Indian Ocean lineage, the latter forming a sequentially paraphyletic assemblage. The nested position of *O. sunia*/*O. insularis *within the Malagasy-Comorian clade, supported by a posterior probability of 0.98 (but not by MP bootstrap percentage), suggests a recent re-colonization of the mainland from an island-distributed ancestor. Yet, we also acknowledge that posterior probabilities could be misleading when short internodes/polytomies are involved [33], which is the case here, and we await the implementations of reversible-jump Markov chain Monte Carlo algorithms to explore these aspects [33].

It is also worth noting that in a similar biogeographic comparison of a different group of birds, non-monophyly of western Indian Ocean taxa was also retrieved by Warren et al. [13]; the Indo-Malayan bulbul *Hypsipetes madagascariensis *was nested within the western Indian Ocean taxa, suggesting a similar scenario to the one we present for *Otus*. Colonization of the mainland from an island distributed ancestor is generally regarded as unlikely because: 1) mainland taxa are considered more competitive than island taxa and, 2) insular populations are smaller and produce fewer emigrants compared to those on continents [34,35]. However, empirical cases of continental re-colonization from islands are accumulating [16,36-38], including cases from Madagascar [39]; this suggests that islands can also act as colonization sources for continental faunas. Data on the phylogeographic structure within the Indo-Malaya *O. sunia *complex may help to decipher which hypothesis (multiple colonizations of the Indian Ocean islands or re-colonization of the mainland) best explains the current pattern. Indeed, if strong and ancient phylogeographic structure occurs within *O. sunia*, we would expect two independent colonizations of the western Indian Ocean islands. In contrast, if genetic data indicate weak differentiation among *O. sunia *populations and patterns of population expansion from western to eastern Indo-Malaya, this would support the hypothesis of re-colonization of the mainland from the Seychelles Islands.

### Vocalisations as a tool to infer evolutionary relationships among scops-owls

The genus *Otus *is fairly homogeneous in plumage relative to many bird genera. However, the Indian Ocean taxa show strong differences from each other in structural, plumage and vocal characters [26,28]. Considering, for example, only the songs of the Comorian taxa (which inhabit islands as close as 50 km from each other), *O. pauliani *gives a very long series of *chaw *notes repeated at about 2/sec; *O. moheliensis *a sequence of hisses; *O. capnodes *a high-pitched whistled *peeooee*; and *O. mayottensis *a series usually of 3–11 deep, single hoots. Intuitively, these differences may be used to argue against close evolutionary relationships; however, our data indicate that the relationship is indeed close in all cases, as could be predicted from the islands' proximity to one another. This study confirms that the vocal and morphological differences are indeed associated with distinct evolutionary lineages, but suggests that they are not related in any simple or obvious way to the evolutionary distance between these lineages, and therefore must be used with caution in identifying affinities between taxa (or lineages). Therefore, we highlight here that the close relationship suggested by Marshall [24] between *O. insularis *and *O. magicus *should be further tested using molecular data, especially if we consider the considerable distance between the two areas (over 6000 km).

### Comparison with the biogeographic history of other avian lineages that colonised the Indian Ocean islands

The geographic and temporal origins of certain western Indian Ocean island bird taxa have received attention in recent years [e.g. [5,9,10,13,16,40-45] this study] (Table 2).

**Table 2:** Summary of divergence dates and geographic origins involving Comorian and/or Malagasy taxa (Note that dating methods and calibration points vary among the studies).

Taxa	Geographic origin	Date estimate	Reference
Philepittinae	Africa or Indo-Malaya	41.2 ± 2.5 mya	[40,41]

Vangidae	Africa or Indo-Malaya	(1) 19.7 mya, (16.8–27.0); (2) 28 ± 4.0 mya	[10,42]

Bernieridae	Africa	25.2 mya (21.4–31.7)	[42]

*Streptopelia picturata*/ *Nesoenas mayeri*	Africa	20.8 ± 4.1 mya	[43]

*Coracina cinerea*	Australasia	18.8 ± 2.2 mya	[16,43]

*Hartlaubius auratus*	Africa or Indo-Malaya	12.9–17 mya	[44]

*Ispidina madagascariensis*	Africa	13.5 ± 2.6 mya	[9,45]

*Corythornis cristata*	Africa	5.5 ± 1.1 mya	[9,45]

Indian Ocean *Dicrurus*	Africa	4.7 mya (95% CI: 2.7–7.4)	[46]

*Motacilla flaviventris*	Africa	4.5 ± 0.3 mya	[47]

*Otus*	Indo-Malaya	3.6 mya (95% HPD: 1.8–6.0)	This study

*Nectarinia souimanga *clade	Africa	1.9–3.9 mya	[48]

*Nectarinia notata *clade	Africa	1.5–3.5 mya	[48]

*Zosterops borbonicus * lineage	Indo-Malaya	1.8 mya	[49]

Indian Ocean *Hypsipetes*	Indo-Malaya	0.6–2.6 mya	[13]

*Zosterops maderaspatanus * lineage	Africa	1.2 mya	[49]

*Anas*	Australasia	No divergence date	[50]

mya: million years ago

Our estimate (3.6 mya, 95% HPD: 1.8–6.0) for the timing of colonization of the Indian Ocean islands by *Otus *coincides with estimates of at least seven other lineages of birds (Table 2), suggesting that the Indian Ocean islands avifauna was highly enriched at that time. The period associated with these multiple independent colonizations corresponds with the emergence of the volcanic islands of the Comoros archipelago (Mohéli 5 mya, Anjouan 11.5–3.9 mya, Mayotte 11.5–7.7 mya and Grande Comore 0.5 mya; [50,51], thus providing possible stepping stones for dispersal between Africa and Madagascar. This possibility is also highlighted by the fact that several species or populations with African biogeographic affinities (*Streptopelia capicola, Turtur tympanistria, Turdus bewsheri*) [53-55], colonized the Comoros islands but the colonization of Madagascar has not yet been achieved.

All faunal exchanges that unambiguously involve Madagascar and Indo-Malaya, or Madagascar and the Seychelles occurred during the last 3.5 mya [[13,49], this study] (note that the biogeographic history of the Philepittinae, Vangidae and the Sturnidae genus *Hartlaubia *are still uncertain or ambiguous). Warren et al. [48] hypothesised for members of the genus *Zosterops *that the colonizations could have been favored by dramatic sea-level shifts that occurred during the last 2.5 mya, which would have allowed the emergence of currently submerged land-masses between the Seychelles and Madagascar [56-58], implying a 'stepping-stone' model. Our divergence dates estimates are slightly older than those of Warren et al. [49], although confidence intervals are largely overlapping. This biogeographic hypothesis fits the three unambiguous described cases involving Madagascar and Indo-Malaya, or Madagascar and the Seychelles (*Hypsipetes*, *Otus *and the *Zosterops borbonicus *lineage).

Subfossil remains of three extinct small owl species have been described from the Mascarene Islands (La Réunion, Mauritius, Rodrigues). Based on certain osteological features, these three species have been included in their own genus, *Mascarenotus*, which has been suggested to be derived from *Otus *[59]. The relationships of *Mascarenotus *with respect to the other owl lineages still needs clarification, as this genus could represent a recent and derived off-shot of the western Indian Ocean lineage, possibly a first off-shot of the colonization process from the Seychelles to more westerly regional islands or even an unrelated lineage of owls.

Whereas the continental biogeographic affinities of certain western Indian Ocean island bird taxa seem largely resolved, the timing of colonizations or faunal affinities among volcanic islands are less well understood and the only aspect that is emerging is the absence of a common pattern, whatever the initial geographic origin. Indeed, all the studies that have been conducted so far indicate explosive diversification and a lack of resolution among the Comoros islands species. These data indicate that once the initial colonization was successful on any of the islands in this archipelago, dispersion and then diversification between nearby islands occurred quickly and randomly. The lack of biogeographic structure at the archipelago scale may thus be partly explained by the geographical arrangement of islands and the short inter-islands distances (maximum distance between two islands in the Comoros archipelago is 90 km) that probably favored colonization by alternative routes.

One final factor that could hide common patterns of diversification is unequal rates of extinction and recolonization across lineages. Indeed, not all lineages would face the same risk of extinction on islands as their characteristics (ecological requirements, population size) often considerably differ. For example, middle-sized birds, like owls (70–120 g), may be more prone to extinction on islands than small birds, like sunbirds (12–15 g) [60]. Yet, even if uneven extinctions rates occurred amongst these lineages, it can be concluded that the overall diversification pattern in the Indian Ocean islands is star-like.

## Conclusion

Our analyses revealed the occurrence of multiple synchronous colonization events of the Indian Ocean islands by scops-owls, at a time when faunistic exchanges involving Madagascar was common as a result of lowered sea-level that would have allowed the formation of stepping-stone islands. Patterns of diversification that emerged from the scops-owls data are: 1) a star-like pattern concerning the order of colonization of the Indian Ocean islands and 2) the high genetic distinctiveness among all Indian Ocean taxa, reinforcing their recognition as distinct species.

## Methods

### Taxonomic sampling

We obtained tissue samples from all western Indian Ocean *Otus *taxa, as well as samples from several Indo-Malayan and Afrotropical species (Table 3), focusing on as many super-species complexes as possible (*sensu *[22]). We obtained tissues for seven of these super-species complexes. We were unable to obtain samples of the distinctive *O. rufescens *and *O. sagittatus*, as well as representatives of four super-species with localised and distant distributions relative to the western Indian Ocean (*brooki*/*angelinae *from Sumatra/Java/Borneo; *mantananensis*/*magicus *from the Lesser Sundas/Philippines/Mollucas; *collari*/*manadensis*/*beccarii *from Sangihe/Sulawesi/Biak and *enganensis*/*alius*/*umbra *from islands off Sumatra). Since the monograph of Marks et al. [22], one further *Otus *species, *O. thilohoffmani *has been described from Sri Lanka [61]. This species is only known in museum collections by the type specimen, deposited in the National Museum Colombo (Sri Lanka), and based on morphology, has been suggested to be related to either *O. rufescens *or *O. spilocephalus *[61]. As a consequence, we did not have access to a tissue sample of this newly described species. With the exception of *O. magicus*, considered by some authors to include *O. insularis *because of similarities in vocalizations [24], none of the species we were unable to sample have been considered closely related to the Indian Ocean taxa [22]. We included two individuals per color morph for *O. rutilus *(sensu [23]). Representatives of the Strigidae genera *Aegolius*, *Athene *and *Glaucidium *(Surniinae), *Asio *(Asioninae), and *Bubo*, *Strix*, New World '*Otus' *(*Megascops*) and the African White-faced Owl ('*Otus' Ptilopsis leucotis*) (Striginae) were included as proximate outgroups. We rooted our trees using sequences from a representative of the Tytonidae (Barn Owl *Tyto alba*), which has been recovered as the sister-group of the Strigidae in molecular and morphological analyses [62,63].

**Table 3:** List of samples used and GenBank accession numbers for the six loci analysed.

Species	Voucher/Tissue number	Geographic	Myoglobin	TGFB2	Cytochrome-*b*	ND2	ATP6	ND3
*Aegolius acadicus*	MVZ 118707 (T)	USA	EU601093	EU600970	U89172	EU601051	EU601160	EU601013
*Asio otus*	MVZ 180184 (T)	USA	EU601097	EU600975	AF082067	EU601055	EU601165	EU601018
*Athene noctua*	MNHN 1995–99 (T)	France	EU601089	EU600966	AJ003948	No sequence	EU601156	EU601009
*Bubo bubo*	MNHN 24–55 (T)	France	EU601069	EU600949	AJ003969	EU601029	EU601137	EU600992
*Bubo virginianus*	MVZ 179340 (T)	USA	EU601092	EU600969	AF168106	EU601050	EU601159	EU601012
*Glaucidium cuculoides*	MNHN 33-9C (JF150, B)	Laos	EU601088	EU600982	No sequence	EU601047	EU601155	No sequence
*Glaucidium gnoma*	MVZ 179345 (T)	USA	EU601094	EU600972	AJ003994	No sequence	EU601162	EU601015
*Otus asio*	MVZ 179828 (T)	USA	EU601096	EU600974	DQ190845	EU601054	EU601164	EU601017
*Otus 'bakkamoena'*	UWBM 67511 (T)	Captive	EU601074	EU600954	EU601110	EU601034	EU601141	EU600997
*Otus capnodes*	MNHN 30-10J (B)	Anjouan	EU601078	EU600957	EU601114	EU601038	EU601145	EU601000
*Otus hartlaubi*	MNHH 32-04G (B)	São Tomé	EU601072	EU600952	EU601108	EU601032	EU601139	EU600995
*Otus hoyi*	ZMUC 114834 (B)	Bolivia	EU601061	EU600942	EU601103	EU601024	EU601130	EU600985
*Otus insularis*	D. Currie 5H21863 (F)	Mahé, Seychelles	EU601059	EU600940	EU601101	EU601022	EU601128	EU600983
*Otus insularis*	D. Currie 5H21866 (F)	Mahé, Seychelles	EU601060	EU600941	EU601102	EU601023	EU601129	EU600984
*Otus ireneae*	MNHN 32-06J (M. Virani C30577) (B)	Kenya	EU601077	EU600956	EU601113	EU601037	EU601144	EU600999
*Otus kenicottii*	MVZ 182896 (T)	USA	EU601095	EU600973	DQ190850	EU601053	EU601163	EU601016
*Otus koepckei*	ZMUC 115283 (B)	Peru	EU601062	EU600943	EU601104	EU601025	EU601131	EU600986
*Otus 'lempiji'*	UWBM 73860 (T)	Captive	EU601076	EU600981	EU601112	EU601036	EU601143	No sequence
*Otus lettia lettia*	MNHN 33-4C (JF142, B)	Laos	EU601073	EU600953	EU601109	EU601033	EU601140	EU600996
*Otus lettia ussuriensis*	UWBM 75379 (T)	Russia	EU601075	EU600955	EU601111	EU601035	EU601142	EU600998
*Otus leucotis*	FMNH 429716 (T)	Congo RD	EU601085	EU600963	EU601120	EU601044	EU601152	EU601006
*Otus longicornis*	FMNH 433020 (T)	Luzon, Philippines	EU601084	EU600962	EU601119	EU601043	EU601151	EU601005
*Otus longicornis*	ZMUC 114206 (B)	Isabela, Philippines	EU601063	No sequence	No sequence	EU601026	EU601132	EU600987
*Otus mayottensis*	MNHN R22 (F)	Mayotte	EU601087	EU600965	EU601122	EU601046	EU601154	EU601008
*Otus megalotis*	FMNH 433019 (T)	Luzon, Philippines	EU601083	EU600961	EU601118	EU601041	EU601150	EU601004
*Otus megalotis*	ZMUC 114208 (B)	Isabela, Philippines	EU601064	EU600944	EU601105	EU601027	EU601133	EU600988
*Otus mirus*	FMNH 357429 (T)	Mindanao, Philippines	EU601099	EU600978	EU601126	EU601057	No sequence	EU601020
*Otus moheliensis*	MNHN E-135 (F)	Mohéli	EU601086	EU600964	EU601121	EU601045	EU601153	EU601007
*Otus pauliani*	MNHN R24 (F)	Grande Comore	EU601100	EU600979	EU601125	EU601058	No sequence	EU601021
*Otus pembaensis*	MNHN uncatalogued (B)	Pemba Island	EU601090	EU600967	EU601123	EU601048	EU601157	EU601010
*Otus pembaensis*	MNHN uncatalogued (B)	Pemba Island	EU601091	EU600968	EU601124	EU601049	EU601158	EU601011
*Otus roboratus*	ZMUC 114634 (B)	Ecuador	EU601065	EU600945	EU601106	EU601028	EU601134	No sequence
*Otus rutilus*	FMNH 393149 (T)	Madagascar	EU601082	EU600960	EF198256	EF198290	EU601149	EU601003
*Otus rutilus*	FMNH 431150/431152 (T)	Madagascar	EU601068	EU600948	EF198273	EF198307	EU601136	EU600991
*Otus rutilus*	FMNH 396240 (T)	Madagascar	EU601066	EU600946	EF198270	EF198304	EU601135	EU600989
*Otus rutilus*	FMNH 427395 (T)	Madagascar	EU601067	EU600947	EF198296	EF198262	No sequence	EU600990
*Otus scops*	MNHN 23-5F (T)	France	EU601079	EU600958	EU601115	EU601039	EU601146	EU601001
*Otus senegalenis*	MVZ uncatalogued (B)	South Africa	EU601098	EU600976, EU600977	EU601127	EU601053	EU601166	EU601019
*Otus spilocephalus*	MNHN 15–58 (B)	China	EU601080	EU600980	EU601116	EU601040	EU601147	No sequence
*Otus sunia*	MNHN 6–98 (B)	Thailand	EU601081	EU600959	EU601117	EU601041	EU601148	EU601002
*Strix aluco*	MNHN CG 1996-114 (T)	France	EU601070	EU600950	EU601107	EU601030	EU601138	EU600993
*Strix woodfordi*	MNHN 32-01H	Kenya	EU601071	EU600951	AJ004066	EU601031	No sequence	EU600994
*Tyto alba*	MVZ 180644	USA	DQ881879	EU600971	AJ004073	EU601052	EU601161	EU601014

B refers to blood, F to feather and T to tissue. Taxonomy follows [22]. Geographic origins of the taxa between quotes are unknown. Acronyms for specimen collections include: FMNH – Field Museum of Natural History, Chicago; MNHN – Muséum National d'Histoire Naturelle, Paris; MVZ, Museum of Vertebrate Zoology, Berkeley; UWBM – University of Washington, Burke Museum, Seattle; ZMUC – Zoological Museum, University of Copenhagen.

### Laboratory procedure

Total DNA was extracted from frozen, EDTA or alcohol preserved tissues (liver, blood, feathers, muscle) using a CTAB-based protocol [64] with an overnight Proteinase K (0,1 mg.ml-1) digestion. ND2, ATP6 and ND3 were amplified and sequenced using primer pairs L5219/H6313 [65], L9245/H9947 [66] and L10755/H11151 [67], respectively. A 900 bp portion of cytochrome-*b *was amplified with the primer pairs L14967-H15487 and L15424-H15916 [23]). Myoglobin intron-2 and TGFB2 intron-5 were amplified with primers Myo2/Myo3F [68,69] and tgf5/tgf6 [70], respectively. The amplification and sequencing protocol were standard [23].

### Phylogenetic analyses

Molecular phylogenies were estimated using parsimony (P) and model-based approaches (maximum likelihood [ML], and Bayesian inferences [BI]), as implemented in PHYML v2.4 [71] and MRBAYES 3.1 [72-74]. Parsimony analyses were conducted with PAUP v4.0b10 [75] using the heuristic tree bisection and reconnection branch-swapping (TBR) algorithm with 100 random addition replicates. Likelihood models were estimated with MRMODELTEST 2.0 [76] using the Akaike Information Criterion [77]. The selected models are listed in Table 4. Clade support in the individual gene trees for the ML and MP analyses was assessed by 1000 non-parametric bootstrap replicates [78]. The six gene regions sequenced differ considerably in their properties and substitution dynamics, as inferred from the parameters of the models (Table 4). Consequently, analyses of concatenated data set were only performed using a mixed-model strategy. The relevance of partitioning the data set by gene and/or codon position was assessed with the Bayes Factor (B_F_) [79,80]. Fourteen partitions (myoglobin intron-2, TGFB2 intron-5, first, second and third codon position of each of the four mitochondrial genes) were considered for the Bayesian concatenated analyses according to the functional properties of the markers. Bayesian analyses for the concatenated data set were performed allowing base frequencies, rate matrix, shape parameter and proportion of invariable sites to vary between the partitions (using the *unlink *and *prset *commands). Between four and six incrementally heated Metropolis-coupled MCMC chains were run for 15 million generations with trees sampled every 100 generations. The first 2*10^6 ^generations (20000 trees) were discarded ('burn-in' period) and the posterior probabilities were estimated from the remaining sampled generations. The default temperature for chain heating (T = 0.2) resulted in not satisfactorily mixing among chains for the concatenated data set; we therefore lowered the temperature to T = 0.05, which resulted in swap frequencies between chains within the 20–70% interval. Two independent Bayesian runs initiated from random starting trees were performed for each data set, and the log-likelihood values and posterior probabilities were checked to ascertain that the chains had reached convergence. We also checked that the Potential Scale Reduction Factor (PSRF) approached 1.0 for all parameters and that the average standard deviation of split frequencies converged towards zero. We detected significant incongruence between the individual gene trees by comparing the topologies and nodal support obtained under different analytical methods (ML, BI). Criteria for incongruence were set at 70% for the bootstrap values [81], and at 0.95 for posterior probabilities [72].

**Table 4:** Model selected and parameters values with their 95% credibility intervals when applicable (obtained with MRBAYES).

	Myoglobin	TGFB2	ND2	ATP6	ND3	**Cytochrome-*b***
Model	K80 + Γ	HKY + Γ	GTR + Γ + I	GTR + Γ + I	GTR + Γ + I	GTR + Γ + I
1^st ^position	NA	NA	GTR + Γ + I	GTR + Γ + I	SYM + Γ	GTR + Γ + I
2^nd ^position	NA	NA	GTR + Γ + I	GTR + Γ + I	GTR + Γ	GTR + Γ + I
3^rd ^position	NA	NA	GTR + Γ + I	GTR + Γ + I	HKY + Γ + I	GTR + Γ
Freq A	0.25	0.25 (0.22–0.28)	0.37 (0.34–0.39)	0.33 (0.30–0.36)	0.32 (0.28–0.36)	0.32 (0.30–0.35)
Freq C	0.25	0.21 (0.18–0.24)	0.41 (0.39–0.43)	0.44 (0.41–0.46)	0.41 (0.37–0.45)	0.44 (0.42–0.47)
Freq G	0.25	0.24 (0.21–0.27)	0.06 (0.05–0.07)	0.07 (0.06–0.08)	0.09 (0.07–0.11)	0.08 (0.07–0.09)
Freq T	0.25	0.30 (0.27–0.33)	0.16 (0.15–0.18)	0.16 (0.14–0.18)	0.18 (0.16–0.21)	0.16 (0.14–0.17)
A-C	NA	NA	0.016 (0.012–0.020)	0.020 (0.013–0.027)	0.022 (0.013–0.032)	0.014 (0.009–0.020)
A-G	NA	NA	0.663 (0.607–0.718)	0.564 (0.484–0.642)	0.535 (0.429–0.637)	0.549 (0.474–0.621)
A-T	NA	NA	0.021 (0.014–0.029)	0.035 (0.022–0.050)	0.015 (0.005–0.029)	0.032 (0.019–0.047)
C-G	NA	NA	0.005 (0.0002–0.014)	0.024 (0.012–0.039)	0.007 (0.0002–0.218)	0.010 (0.0002–0.020)
C-T	NA	NA	0.232 (0.188–0.281)	0.323 (0.252–0.399)	0.327 (0.240–0.425)	0.321 (0.259–0.390)
G-T	NA	NA	0.062 (0.035–0.094)	0.035 (0.010–0.069)	0.094 (0.051–0.146)	0.074 (0.041–0.114)
Γ	0.10 (0.09–.12)	0.586 (0.382–0.936)	0.997 (0.826–1.195)	0.901 (0.676–1.158)	0.910 (0.604–1.295)	0.869 (0.702–1.069)
I	NA	NA	0.338 (0.302–0.374)	0.400 (0.345–0.447)	0.368 (0.287–0.433)	0.465 (0.426–0.501)
Ts/Tv	4.24 (3.08–5.75)	5.90 (4.32–7.795)	NA	NA	NA	NA
-ln (ML)	2198.81	2131.71	10556.45	6550.30	3266.29	8623.79
-ln (BI)	2442.98	2179.36	10582.97	6585.41	3305.87	8658.36
-ln (BI partitioned)	NA	NA	10161.23	6232.87	3146.47	8128.06

NA means not applicable. Reported likelihood scores for the BI refer to the arithmetic mean.

### Molecular dating analyses

Owls have a very rich fossil record [22,82], yet, the taxonomic history of most of these taxa remains controversial and, to our knowledge, no cladistic analyses including fossil and modern taxa has been conducted to date. This hampers the use of most of these fossils as calibration points in our analyses. The family Strigidae is usually divided into three subfamilies or tribes: Striginae, Surniinae and Asioninae [21]. These three subfamilies are defined by a combination of shared characters [[82]* fide *[83]]. The least inclusive of these three subfamilies, the Asioninae, consists of three genera and nine species (two of the genera, *Pseudoscops *and *Nesasio *are endemic to Jamaica and the Solomon Islands, respectively, whereas *Asio *is widely distributed). The Asionae differ osteologically from other owls by having: 1) the anterior rim of the internal trochlea not protruding more anteriorly than the anterior rim of the external trochlea, 2) the external calcaneal ridge bent posteriorly, 3) bony loop broad and 4) tubercle for *Musculus tibialis antiquus *displaced externally [83]. The most ancient fossil having this combination of characters *Intulula tinnipara*, has been dated from the Early Miocene (23.7–16.4 mya) [84]. We therefore used this date as a minimum age for the split between *Asio otus *(the member of the Asioninae we sampled) and its closest relative. As a second calibration point, we used the split between *O. pauliani *(endemic to Grande Comore, a volcanic island) and its closest relative at 0.5 mya. This date corresponds to the emergence of Grande Comore [51], and is thus the oldest possible age for the colonization of the *O. pauliani *lineage. We used these two calibration points in combination.

We used BEAST V1.4.6 [85-87] to estimate the divergence dates within the genus *Otus*. We assigned the best fitting model, as estimated by MRMODELTEST2, to each of the six loci. We used an exponential distribution for the fossil calibration bound [88]. We set the lower bound of the exponential distribution to 16.4 mya, which correspond to the lowest bound of the Early Miocene epoch and the exponential mean to 2.5, so that the 95% distribution probability fell within the 23.7–16.4 mya interval, corresponding to the Early Miocene, which is the epoch for the first Asioninae fossil. For the geological calibration point (emergence of Grande Comore), we used a normal distribution with the mean and standard deviation set to 0.5 mya and 0.1 mya, respectively. We assumed a Yule Speciation Process for the tree prior and an Uncorrelated Lognormal distribution for the molecular clock model [86]. We used default prior distributions for all other parameters and ran MCMC chains for 75 million generations, as the effective sample size for some parameter estimates was not large enough using the default length (10 million generations).

## Authors' contributions

JF did part of the laboratory work, edited and aligned the sequences, performed the phylogenetic and dating analyses and wrote the first draft of the manuscript. JMP did part of the laboratory work and helped to draft the manuscript. SMG provided tissue samples for the study, wrote several sections of the manuscript and commented on multiple versions. VB collected tissue samples in the field and commented on the manuscript. MM assisted with sample collection and with the draft of the manuscript. RCKB helped with tissue sample acquisition, provided funding and commented on multiple versions of the manuscript. DC acquired field samples. RS acquired field samples, advised on taxon sampling in relation to morphology and vocalizations, and helped to prepare and finalize the manuscript. MZV collected blood samples and commented on the manuscript. ST helped with collecting samples. AH helped with capturing *Otus pembaensis*. CC did part of the laboratory work. EP collected tissue samples in the field and commented on the manuscript. All authors have read and approved the final version of the manuscript.

## Supplementary Material

Additional File 1 Fifty percent majority-rule consensus tree (arithmetic mean -ln = 2442.98) obtained from the Bayesian Inference analyses of myoglobin intron-2 (749 bp). Values next to branches represent MP/ML bootstrap percentages (below) and BI posterior probabilities (above). Gray blocks represent the Indian Ocean taxa. Species between quotes indicate samples for which geographic origin is unknown (captive individuals). The phylogram represents the relationships among owls as inferred from myoglobin intron-2 sequence data. Click here for file

Additional File 2 Fifty percent majority-rule consensus tree (arithmetic mean -ln = 3427.03) obtained from the Bayesian Inference analyses TGFB2 intron-5 (602 bp). Values next to branches represent MP/ML bootstrap percentages (below) and BI posterior probabilities (above). Gray blocks represent the Indian Ocean taxa. Species between quotes indicate samples for which geographic origin is unknown (captive individuals). The phylogram represents the relationships among owls as inferred from TGFB2 intron-5 sequence data. Click here for file

Additional File 3 Fifty percent majority-rule consensus tree (arithmetic mean -ln = 10161.23) obtained from the Bayesian Inference analyses of the mitochondrial ND2 gene (1041 bp) under a mixed-model strategy (partitioned by codon position). Values next to branches represent MP/ML bootstrap percentages (below) and BI posterior probabilities (above). Gray blocks represent the Indian Ocean taxa. Species between quotes indicate samples for which geographic origin is unknown (captive individuals). The phylogram represents the relationships among owls as inferred from ND2 sequence data. Click here for file

Additional File 4 Fifty percent majority-rule consensus tree (arithmetic mean -ln = 6232.87) obtained from the Bayesian Inference analyses of the mitochondrial ATP6 gene (684 bp) under a mixed-model strategy (partitioned by codon position). Values next to branches represent MP/ML bootstrap percentages (below) and BI posterior probabilities (above). Gray blocks represent the Indian Ocean taxa. Species between quotes indicate samples for which geographic origin is unknown (captive individuals). The phylogram represents the relationships among owls as inferred from ATP6 sequence data. Click here for file

Additional File 5 Fifty percent majority-rule consensus tree (arithmetic mean -ln = 3146.47) obtained from the Bayesian Inference analyses of the mitochondrial ND3 gene (351 bp) under a mixed-model strategy (partitioned by codon position). Values next to branches represent MP/ML bootstrap percentages (below) and BI posterior probabilities (above). Gray blocks represent the Indian Ocean taxa. Species between quotes indicate samples for which geographic origin is unknown (captive individuals). The phylogram represents the relationships among owls as inferred from ND3 sequence data. Click here for file

Additional File 6 Fifty percent majority-rule consensus tree (arithmetic mean -ln = 8128.06) obtained from the Bayesian Inference analyses of the mitochondrial Cytochrome-*b *gene (1041 bp) under a mixed-model strategy (partitioned by codon position). Values next to branches represent MP/ML bootstrap percentages (below) and BI posterior probabilities (above). Gray blocks represent the Indian Ocean taxa. Species between quotes indicate samples for which geographic origin is unknown (captive individuals). The phylogram represents the relationships among owls as inferred from Cytochrome-*b *sequence data. Click here for file
