# Supplementary figures and images for: Tracing the colonization history of the Indian Ocean scops-owls (Strigiformes: Otus) with further insight into the spatio-temporal origin of the Malagasy avifauna
Source: BMC Evol Biol. 2008 Jul 9;8:197. doi: 10.1186/1471-2148-8-197 (PMC2483963; doi:10.1186/1471-2148-8-197)

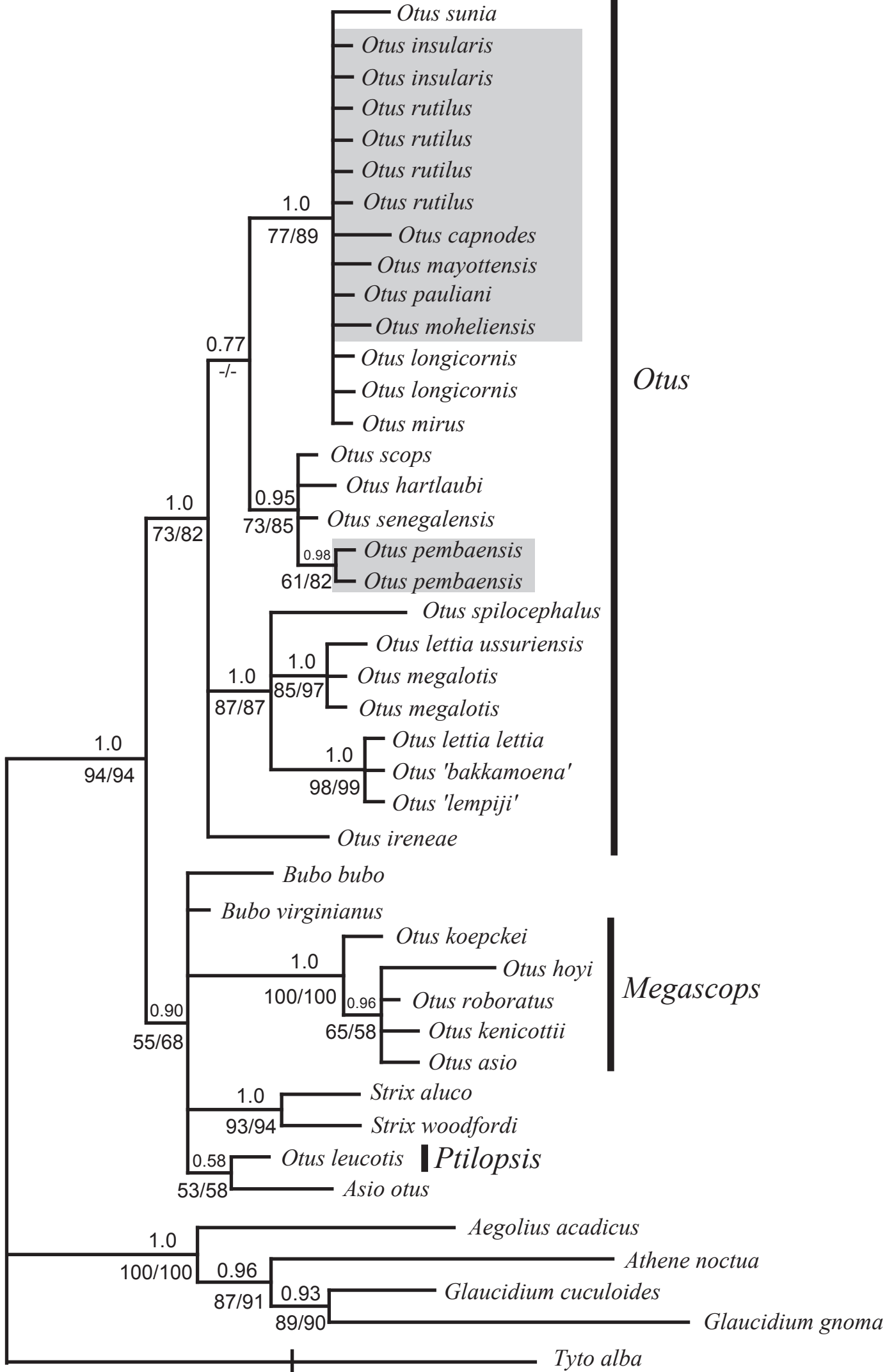

Supplement: Additional File 1 — Fifty percent majority-rule consensus tree (arithmetic mean -ln = 2442.98) obtained from the Bayesian Inference analyses of myoglobin intron-2 (749 bp). Values next to branches represent MP/ML bootstrap percentages (below) and BI posterior probabilities (above). Gray blocks represent the Indian Ocean taxa. Species between quotes indicate samples for which geographic origin is unknown (captive individuals). The phylogram represents the relationships among owls as inferred from myoglobin intron-2 sequence data. [file 1471-2148-8-197-S1.pdf]

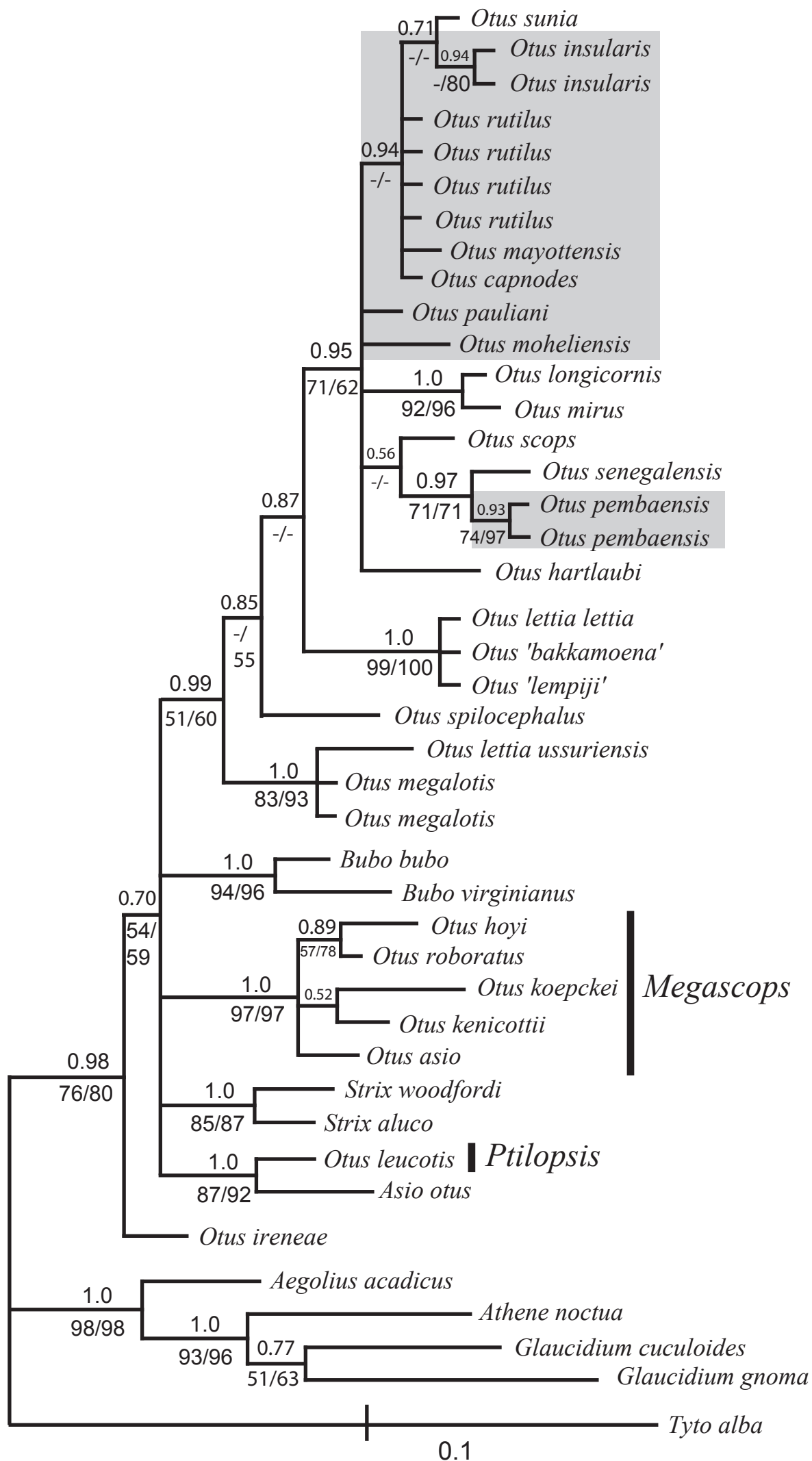

Supplement: Additional File 2 — Fifty percent majority-rule consensus tree (arithmetic mean -ln = 3427.03) obtained from the Bayesian Inference analyses TGFB2 intron-5 (602 bp). Values next to branches represent MP/ML bootstrap percentages (below) and BI posterior probabilities (above). Gray blocks represent the Indian Ocean taxa. Species between quotes indicate samples for which geographic origin is unknown (captive individuals). The phylogram represents the relationships among owls as inferred from TGFB2 intron-5 sequence data. [file 1471-2148-8-197-S2.pdf]

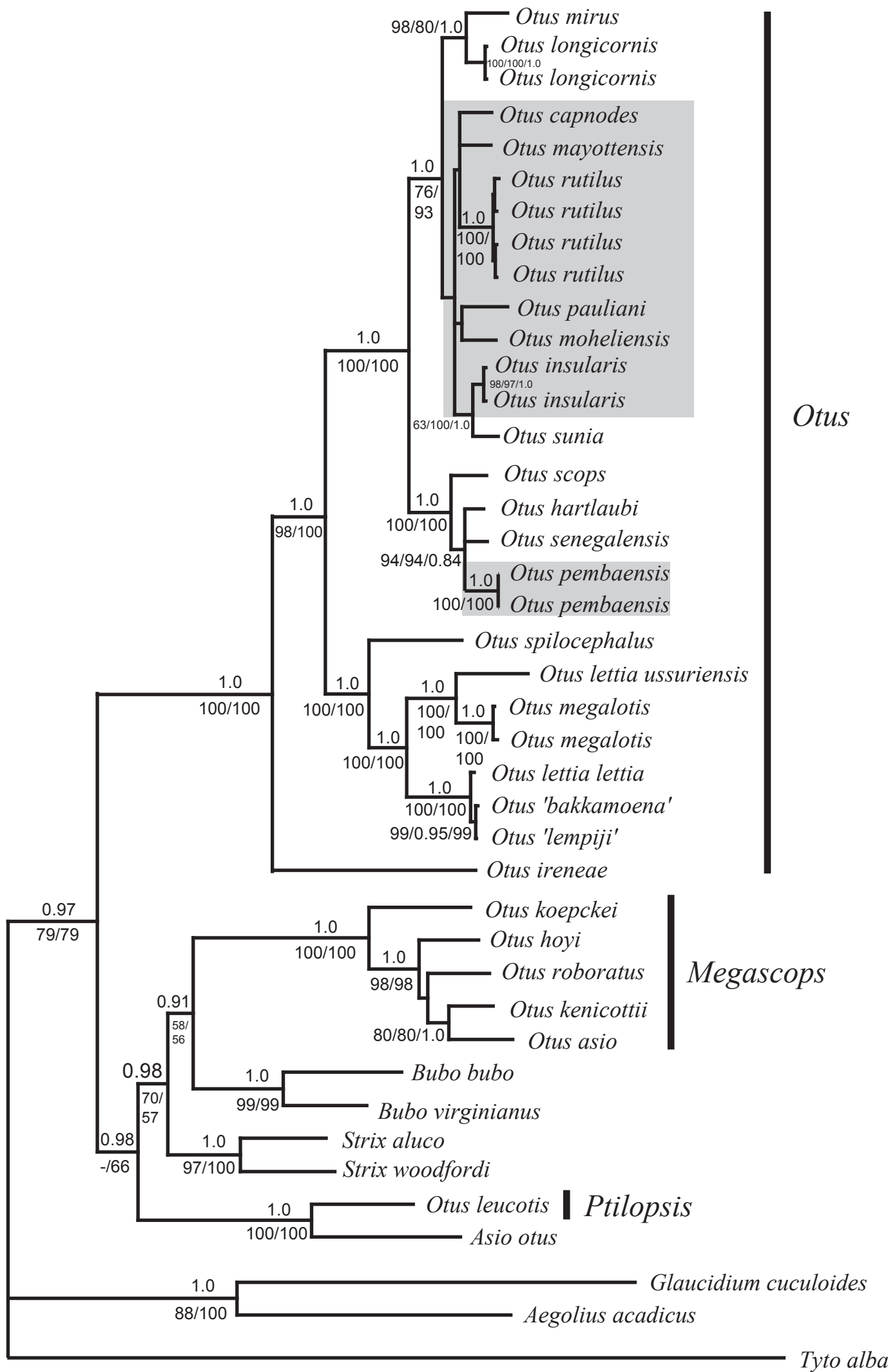

0.1

Supplement: Additional File 3 — Fifty percent majority-rule consensus tree (arithmetic mean -ln = 10161.23) obtained from the Bayesian Inference analyses of the mitochondrial ND2 gene (1041 bp) under a mixed-model strategy (partitioned by codon position). Values next to branches represent MP/ML bootstrap percentages (below) and BI posterior probabilities (above). Gray blocks represent the Indian Ocean taxa. Species between quotes indicate samples for which geographic origin is unknown (captive individuals). The phylogram represents the relationships among owls as inferred from ND2 sequence data. [file 1471-2148-8-197-S3.pdf]

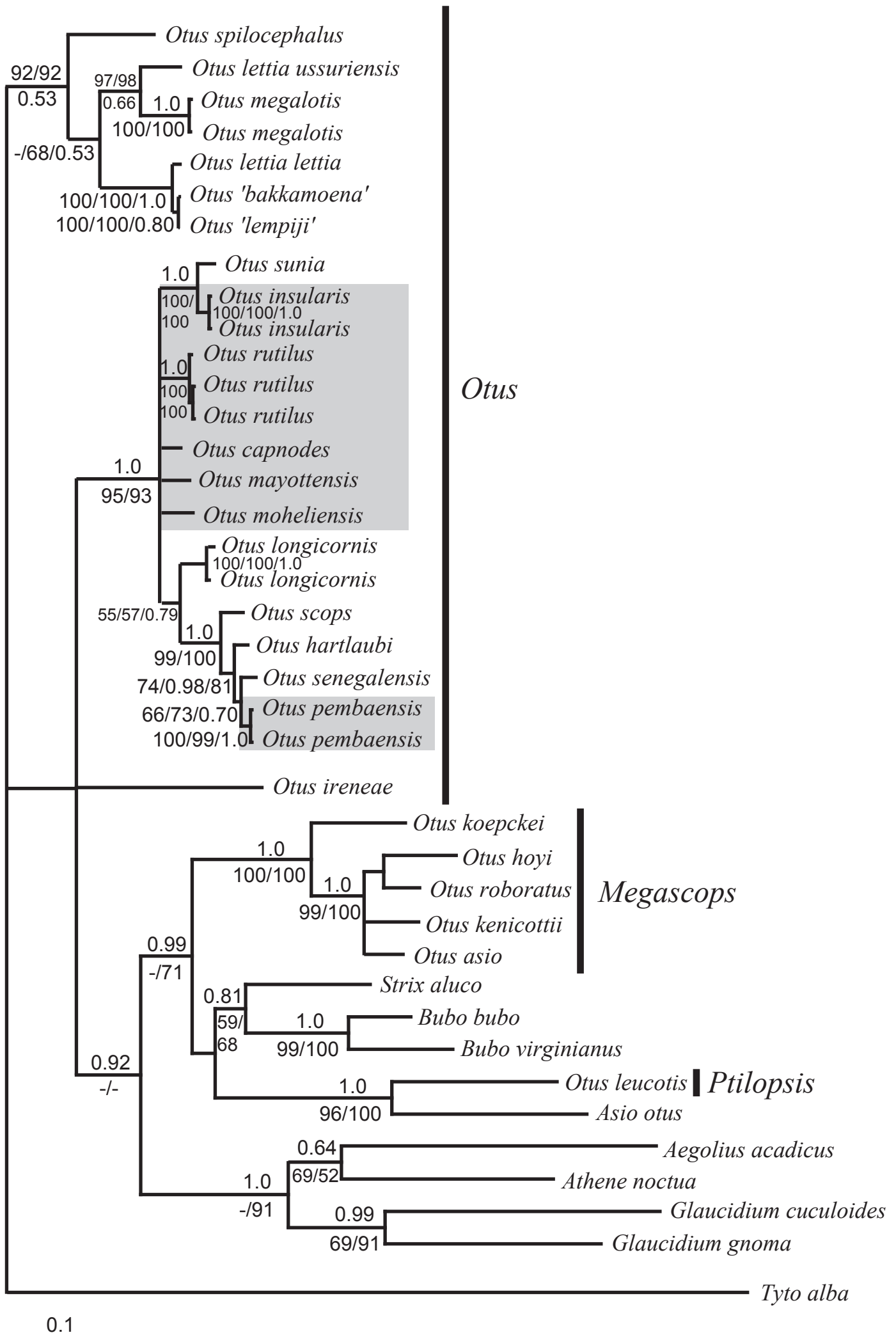

Supplement: Additional File 4 — Fifty percent majority-rule consensus tree (arithmetic mean -ln = 6232.87) obtained from the Bayesian Inference analyses of the mitochondrial ATP6 gene (684 bp) under a mixed-model strategy (partitioned by codon position). Values next to branches represent MP/ML bootstrap percentages (below) and BI posterior probabilities (above). Gray blocks represent the Indian Ocean taxa. Species between quotes indicate samples for which geographic origin is unknown (captive individuals). The phylogram represents the relationships among owls as inferred from ATP6 sequence data. [file 1471-2148-8-197-S4.pdf]

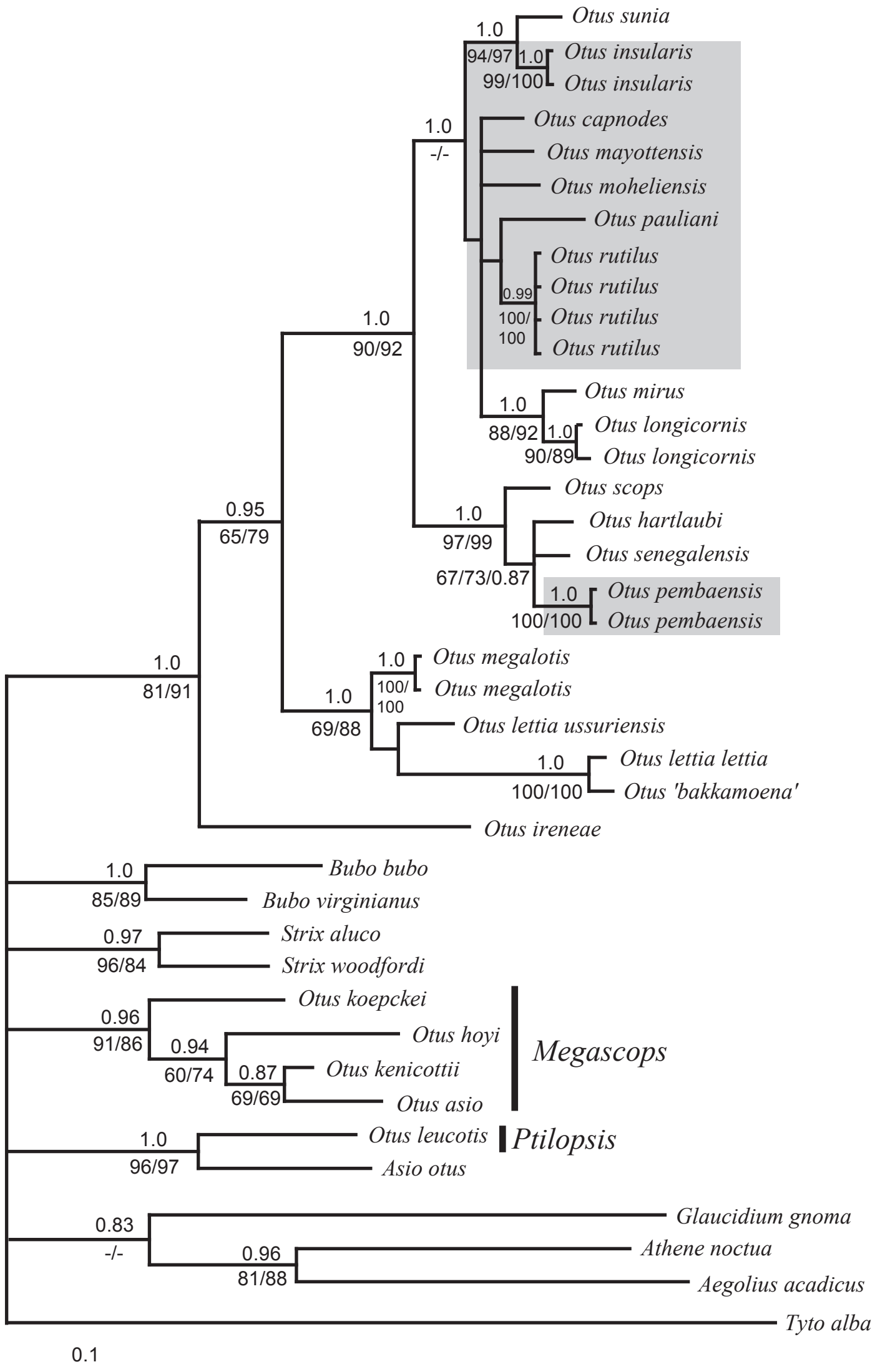

Supplement: Additional File 5 — Fifty percent majority-rule consensus tree (arithmetic mean -ln = 3146.47) obtained from the Bayesian Inference analyses of the mitochondrial ND3 gene (351 bp) under a mixed-model strategy (partitioned by codon position). Values next to branches represent MP/ML bootstrap percentages (below) and BI posterior probabilities (above). Gray blocks represent the Indian Ocean taxa. Species between quotes indicate samples for which geographic origin is unknown (captive individuals). The phylogram represents the relationships among owls as inferred from ND3 sequence data. [file 1471-2148-8-197-S5.pdf]

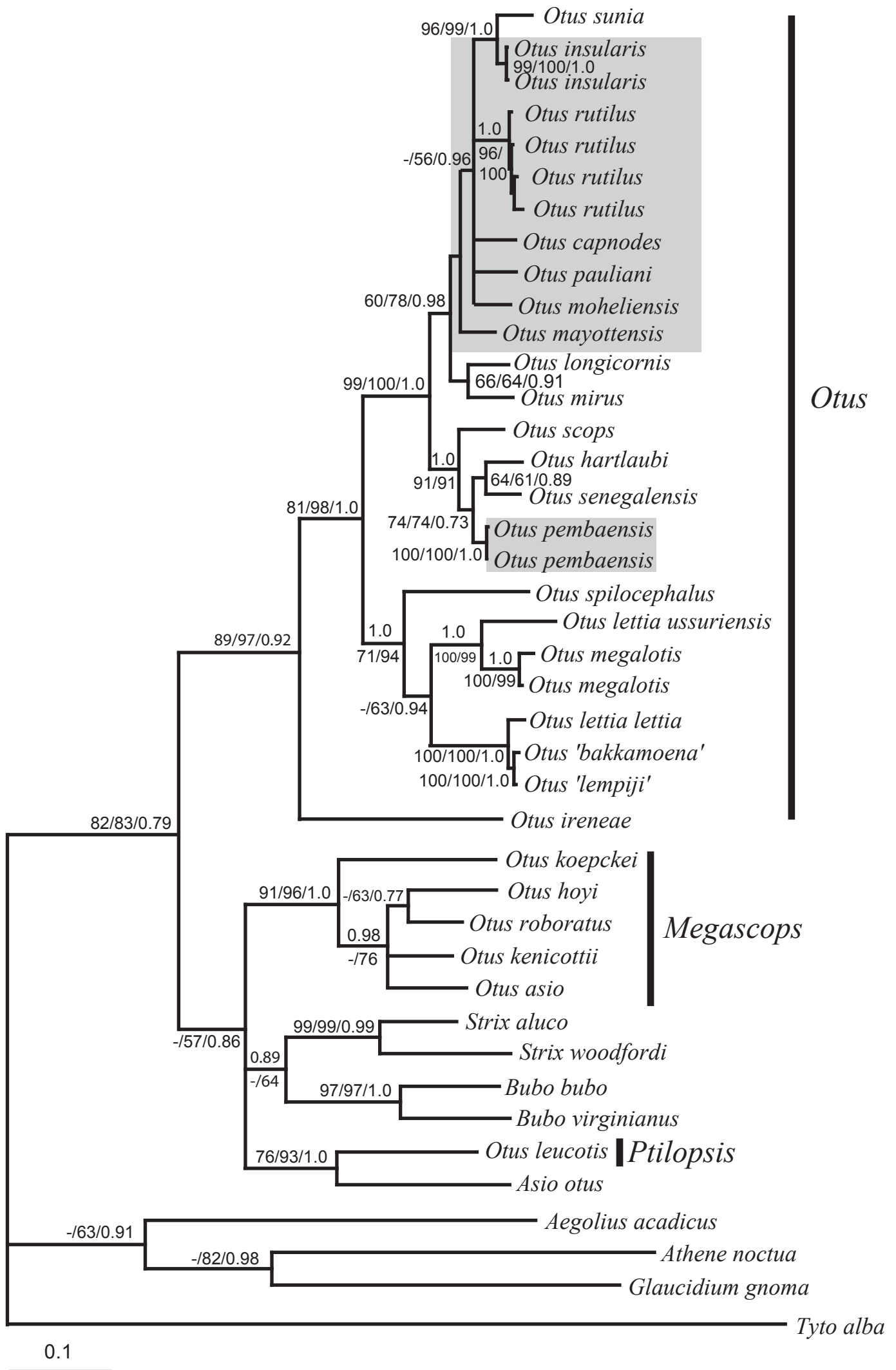

Supplement: Additional File 6 — Fifty percent majority-rule consensus tree (arithmetic mean -ln = 8128.06) obtained from the Bayesian Inference analyses of the mitochondrial Cytochrome-b gene (1041 bp) under a mixed-model strategy (partitioned by codon position). Values next to branches represent MP/ML bootstrap percentages (below) and BI posterior probabilities (above). Gray blocks represent the Indian Ocean taxa. Species between quotes indicate samples for which geographic origin is unknown (captive individuals). The phylogram represents the relationships among owls as inferred from Cytochrome-b sequence data. [file 1471-2148-8-197-S6.pdf]
